# *catena*-Poly[[aqua­{4-[*N*′-(2,4-dioxo-3-pentyl­idene)­hydrazino]­benzoato}­copper(II)]-μ-acetato]

**DOI:** 10.1107/S1600536808016073

**Published:** 2008-06-19

**Authors:** Lujiang Hao, Chunhua Mu, Ridong Wang

**Affiliations:** a College of Food and Biological Engineering, Shandong Institute of Light Industry, Jinan, 250353, People’s Republic of China; b Maize Research Insitute, Shandong Academy of Agricultural Science, Jinan, 250100, People’s Republic of China; c Department of Clinical Medicine, Medical School, Shandong University, Jinan, 250012, People’s Republic of China

## Abstract

In the title compound, [Cu(CH_3_CO_2_)(C_12_H_11_N_2_O_4_)(H_2_O)]_*n*_, the Cu^II^ cation is tetra­coordinated by three carboxyl­ate O atoms from one 4-[*N*′-(2,4-dioxo-3-pentyl­idene)­hydrazino]­benzoate ligand and two acetate bridges, and by one water mol­ecule. The acetate bridges link adjacent Cu^II^ cations, forming a chain. The crystal structure involves O—H⋯O hydrogen bonds.

## Related literature

For uses of carboxylic acids in materials science, see: Church & Halvorson (1959 ▶). For uses in biological systems, see: Chung *et al.* (1971 ▶); Okabe & Oya (2000 ▶); Serre *et al.* (2005 ▶); Pocker & Fong (1980 ▶); Scapin *et al.* (1997 ▶); Kim *et al.* (2001 ▶).
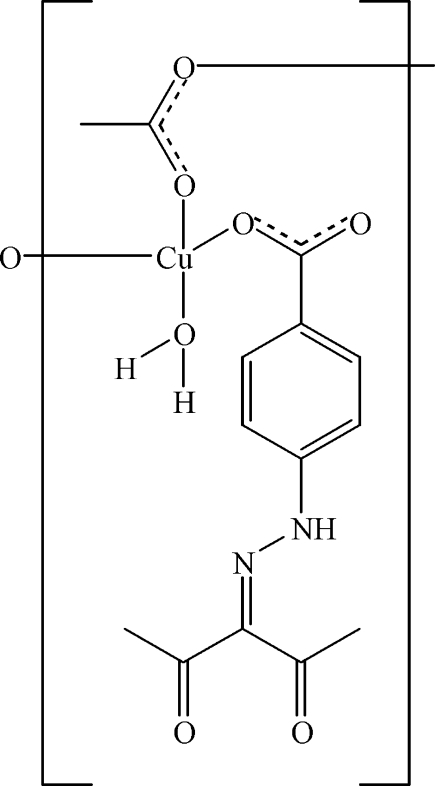


## Experimental

### 

#### Crystal data

[Cu(C_2_H_3_O_2_)(C_12_H_11_N_2_O_4_)(H_2_O)]
*M* *_r_* = 387.83
Monoclinic, 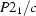

*a* = 8.106 (2) Å
*b* = 23.918 (4) Å
*c* = 8.946 (2) Å
β = 106.90 (3)°
*V* = 1659.5 (6) Å^3^
*Z* = 4
Mo *K*α radiation
μ = 1.35 mm^−1^
*T* = 293 (2) K
0.43 × 0.28 × 0.22 mm

#### Data collection

Bruker APEXII CCD diffractometer
Absorption correction: multi-scan (*SADABS*; Bruker, 2004 ▶) *T*_min_ = 0.594, *T*_max_ = 0.755
8654 measured reflections
4235 independent reflections
2693 reflections with *I* > 2σ(*I*)
*R*_int_ = 0.034

#### Refinement

*R*[*F* ^2^ > 2σ(*F* ^2^)] = 0.064
*wR*(*F* ^2^) = 0.219
*S* = 1.00
4235 reflections
226 parameters
3 restraints
H atoms treated by a mixture of independent and constrained refinement
Δρ_max_ = 0.94 e Å^−3^
Δρ_min_ = −1.73 e Å^−3^

### 

Data collection: *APEX2* (Bruker, 2004 ▶); cell refinement: *SAINT-Plus* (Bruker, 2004 ▶); data reduction: *SAINT-Plus*; program(s) used to solve structure: *SHELXS97* (Sheldrick, 2008 ▶); program(s) used to refine structure: *SHELXL97* (Sheldrick, 2008 ▶); molecular graphics: *SHELXTL* (Sheldrick, 2008 ▶); software used to prepare material for publication: *SHELXTL*.

## Supplementary Material

Crystal structure: contains datablocks global, I. DOI: 10.1107/S1600536808016073/cf2197sup1.cif

Structure factors: contains datablocks I. DOI: 10.1107/S1600536808016073/cf2197Isup2.hkl

Additional supplementary materials:  crystallographic information; 3D view; checkCIF report

## Figures and Tables

**Table 1:** Hydrogen-bond geometry (Å, °)

*D*—H⋯*A*	*D*—H	H⋯*A*	*D*⋯*A*	*D*—H⋯*A*
N1—H1⋯O2	0.94 (5)	1.90 (5)	2.617 (5)	132 (4)
O7—H7*A*⋯O1^ii^	0.84 (4)	1.91 (4)	2.739 (4)	168 (5)
O7—H7*B*⋯O4^iii^	0.84 (5)	2.00 (3)	2.774 (5)	153 (6)

Symmetry codes: (ii) 
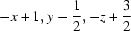
; (iii) 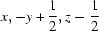.

**Fig. 1.**
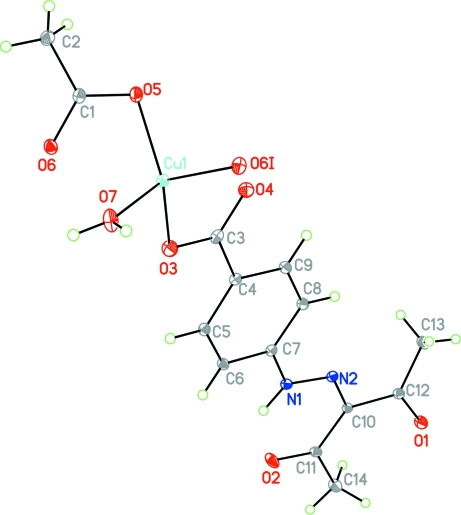
A segment of the polymeric structure of (I), showing the atomic numbering scheme and 30% probability displacement ellipsoids. [Symmetry code: (I), x, 3/2 - y, z - 1/2.]

**Fig. 2.**
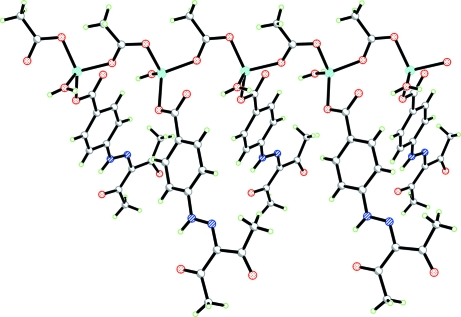
One-dimensional chain of (I).

**Fig. 3.**
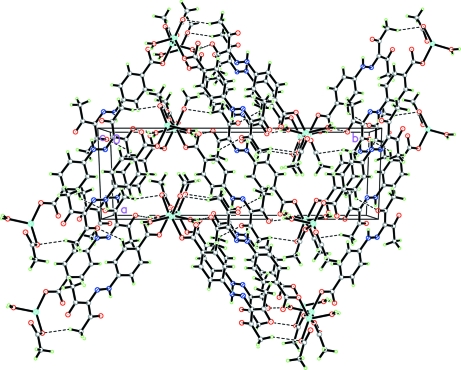
The packing of (I), with hydrogen bonds shown as dashed lines.

## supplementary crystallographic information

### Comment

In recent years, carboxylates have been widely used as polydentate ligands, which can coordinate to transition or rare earth ions yielding complexes with interesting properties that are useful in materials science (Church & Halvorson, 1959; Chung *et al.*, 1971) and in biological systems (Okabe & Oya, 2000; Serre *et al.*, 2005; Pocker & Fong, 1980; Scapin *et al.*, 1997). For example, Kim *et al.* (2001) focused on the syntheses of transition metal complexes containing benzenecarboxylate and rigid aromatic pyridine ligands in order to study their electronic conductivity and magnetic properties. The importance of transition metal dicarboxylate complexes motivated us to pursue synthetic strategies for these compounds, using sodium 4-(2-(diacetylmethylene)hydrazino)benzoate as a polydentate ligand. Here we report the synthesis and X-ray crystal structure analysis of the title compound. The asymmetric unit of the title compound is shown in Fig. 1. The copper(II) cation is tetracoordinated by three carboxylate oxygen atoms from one 2,4-dioxo-3-pentylidene)hydrazino]benzoate ligand and two acetate bridges, and by one water molecule. The acetate bridges link adjacent copper(II) cations, forming a chain, shown in Fig. 2. The Cu—O bond distances are in the range 1.970 (4)–2.031 (4) Å. The packing involves O—H···O hydrogen bonds, with O···O in the range 2.744 (5)–3.058 (6) Å, as shown in Fig. 3.

### Experimental

A mixture of copper(II) acetate (0.5 mmol), 4-(2-(diacetylmethylene)hydrazino)benzoic acid (0.5 mmol), water (8 ml) and ethanol (8 ml) in a 25 ml Teflon-lined stainless steel autoclave was kept at 413 K for three days. Colorless crystals were obtained after cooling to room temperature with a yield of 27%. Anal. Calc. for C_14_H_15_CuN_2_O_7_: C 43.43, H 3.88, N 7.24%; Found: C 43.36, H 3.79, N 7.16%.

### Refinement

The H atoms of the water molecule were located in a difference density map and were refined with distance restraints H···H = 1.38 (1) Å, O—H = 0.84 (1) Å, and with *U*_iso_(H) = 1.2*U*_eq_(O). The N-bound H atom was also located in a map, and was refined with no positional restraints and with *U*_iso_(H) = 1.2*U*_eq_(N). All other H atoms were placed in calculated positions with a C—H bond distance of 0.93 Å and *U*_iso_(H) = 1.2*U*_eq_(C).

### Crystal data

**Table:**

[Cu(C_2_H_3_O_2_)(C_12_H_11_N_2_O_4_)(H_2_O)]	*F*_000_ = 796
*M**_r_* = 387.83	*D*_x_ = 1.552 Mg m^−^^3^
Monoclinic, *P*2_1_/*c*	Mo *K*α radiation λ = 0.71073 Å
Hall symbol: -P 2ybc	Cell parameters from 4235 reflections
*a* = 8.106 (2) Å	θ = 1.7–28.8º
*b* = 23.918 (4) Å	µ = 1.35 mm^−^^1^
*c* = 8.946 (2) Å	*T* = 293 (2) K
β = 106.90 (3)º	Block, blue
*V* = 1659.5 (6) Å^3^	0.43 × 0.28 × 0.22 mm
*Z* = 4	

### Data collection

**Table:**

Bruker APEXII CCD diffractometer	4235 independent reflections
Radiation source: fine-focus sealed tube	2693 reflections with *I* > 2σ(*I*)
Monochromator: graphite	*R*_int_ = 0.034
*T* = 293(2) K	θ_max_ = 28.8º
φ and ω scans	θ_min_ = 1.7º
Absorption correction: multi-scan(SADABS; Bruker, 2004)	*h* = −10→9
*T*_min_ = 0.594, *T*_max_ = 0.755	*k* = −31→29
8654 measured reflections	*l* = −8→11

### Refinement

**Table:**

Refinement on *F*^2^	Secondary atom site location: difference Fourier map
Least-squares matrix: full	Hydrogen site location: inferred from neighbouring sites
*R*[*F*^2^ > 2σ(*F*^2^)] = 0.064	H atoms treated by a mixture of independent and constrained refinement
*wR*(*F*^2^) = 0.219	*w* = 1/[σ^2^(*F*_o_^2^) + (0.167*P*)^2^] where *P* = (*F*_o_^2^ + 2*F*_c_^2^)/3
*S* = 1.00	(Δ/σ)_max_ < 0.001
4235 reflections	Δρ_max_ = 0.94 e Å^−^^3^
226 parameters	Δρ_min_ = −1.73 e Å^−^^3^
3 restraints	Extinction correction: none
Primary atom site location: structure-invariant direct methods	

### Special details

**Table:**

Geometry. All e.s.d.'s (except the e.s.d. in the dihedral angle between two l.s. planes) are estimated using the full covariance matrix. The cell e.s.d.'s are taken into account individually in the estimation of e.s.d.'s in distances, angles and torsion angles; correlations between e.s.d.'s in cell parameters are only used when they are defined by crystal symmetry. An approximate (isotropic) treatment of cell e.s.d.'s is used for estimating e.s.d.'s involving l.s. planes.
Refinement. Refinement of *F*^2^ against ALL reflections. The weighted *R*-factor *wR* and goodness of fit *S* are based on *F*^2^, conventional *R*-factors *R* are based on *F*, with *F* set to zero for negative *F*^2^. The threshold expression of *F*^2^ > σ(*F*^2^) is used only for calculating *R*-factors(gt) *etc*. and is not relevant to the choice of reflections for refinement. *R*-factors based on *F*^2^ are statistically about twice as large as those based on *F*, and *R*- factors based on ALL data will be even larger.

### Fractional atomic coordinates and isotropic or equivalent isotropic displacement parameters (Å^2^)

**Table:**

	*x*	*y*	*z*	*U*_iso_*/*U*_eq_	
Cu1	−0.04150 (6)	0.25987 (2)	0.28607 (6)	0.0311 (2)	
C1	−0.2534 (6)	0.28239 (16)	−0.0029 (5)	0.0339 (8)	
C2	−0.4039 (6)	0.2997 (2)	−0.1400 (5)	0.0490 (11)	
H2A	−0.3776	0.2918	−0.2358	0.073*	
H2B	−0.4245	0.3390	−0.1337	0.073*	
H2C	−0.5049	0.2793	−0.1375	0.073*	
C3	0.1783 (5)	0.33727 (16)	0.4174 (5)	0.0340 (8)	
C4	0.3381 (5)	0.37225 (15)	0.4810 (4)	0.0290 (8)	
C5	0.4965 (5)	0.36320 (16)	0.4439 (5)	0.0326 (8)	
H5A	0.5036	0.3340	0.3773	0.039*	
C6	0.6407 (5)	0.39724 (16)	0.5056 (5)	0.0331 (8)	
H6A	0.7423	0.3912	0.4796	0.040*	
C7	0.6292 (5)	0.43991 (15)	0.6058 (4)	0.0299 (8)	
C8	0.4760 (5)	0.44926 (16)	0.6466 (5)	0.0333 (8)	
H8A	0.4712	0.4778	0.7158	0.040*	
C9	0.3298 (5)	0.41525 (16)	0.5824 (5)	0.0332 (8)	
H9A	0.2283	0.4217	0.6082	0.040*	
C10	0.9194 (5)	0.54195 (16)	0.8363 (4)	0.0281 (7)	
C11	1.0841 (5)	0.53851 (17)	0.7897 (5)	0.0346 (8)	
C12	0.8959 (5)	0.58020 (16)	0.9595 (5)	0.0339 (8)	
C13	0.7155 (6)	0.5879 (3)	0.9759 (7)	0.0562 (14)	
H13A	0.7201	0.6133	1.0600	0.084*	
H13B	0.6720	0.5524	0.9978	0.084*	
H13C	0.6409	0.6027	0.8804	0.084*	
C14	1.2423 (6)	0.5712 (2)	0.8705 (6)	0.0480 (11)	
H14A	1.3319	0.5631	0.8233	0.072*	
H14B	1.2801	0.5610	0.9790	0.072*	
H14C	1.2164	0.6104	0.8612	0.072*	
N1	0.7769 (4)	0.47437 (13)	0.6639 (4)	0.0306 (7)	
H1	0.872 (6)	0.471 (2)	0.625 (5)	0.037*	
N2	0.7779 (4)	0.51093 (13)	0.7725 (4)	0.0307 (7)	
O1	1.0199 (4)	0.60409 (14)	1.0486 (4)	0.0493 (8)	
O2	1.0905 (4)	0.50681 (17)	0.6823 (5)	0.0599 (11)	
O3	0.1881 (4)	0.29684 (14)	0.3292 (4)	0.0511 (8)	
O4	0.0403 (4)	0.34837 (14)	0.4497 (4)	0.0456 (8)	
O5	−0.2621 (4)	0.28883 (13)	0.1365 (3)	0.0411 (7)	
O6	−0.1154 (5)	0.26235 (12)	−0.0240 (4)	0.0424 (8)	
O7	0.0168 (6)	0.18300 (14)	0.2414 (4)	0.0566 (10)	
H7A	0.019 (8)	0.1569 (15)	0.305 (5)	0.068*	
H7B	0.056 (7)	0.172 (2)	0.169 (4)	0.068*	

### Atomic displacement parameters (Å^2^)

**Table:**

	*U*^11^	*U*^22^	*U*^33^	*U*^12^	*U*^13^	*U*^23^
Cu1	0.0339 (4)	0.0265 (3)	0.0328 (4)	−0.00674 (17)	0.0093 (2)	−0.00117 (17)
C1	0.046 (2)	0.0210 (17)	0.033 (2)	0.0006 (15)	0.0104 (18)	−0.0006 (15)
C2	0.051 (3)	0.052 (3)	0.038 (2)	0.014 (2)	0.005 (2)	0.001 (2)
C3	0.040 (2)	0.0279 (19)	0.031 (2)	−0.0096 (15)	0.0056 (17)	0.0025 (15)
C4	0.0317 (18)	0.0214 (16)	0.0314 (19)	−0.0037 (13)	0.0054 (15)	0.0024 (14)
C5	0.038 (2)	0.0241 (17)	0.034 (2)	−0.0007 (15)	0.0091 (16)	−0.0046 (15)
C6	0.034 (2)	0.0295 (19)	0.037 (2)	0.0014 (15)	0.0118 (17)	−0.0041 (16)
C7	0.034 (2)	0.0225 (17)	0.031 (2)	−0.0020 (14)	0.0057 (16)	0.0002 (14)
C8	0.034 (2)	0.0267 (18)	0.038 (2)	−0.0027 (14)	0.0083 (17)	−0.0081 (15)
C9	0.033 (2)	0.0284 (19)	0.040 (2)	−0.0043 (15)	0.0126 (17)	−0.0059 (16)
C10	0.0255 (17)	0.0255 (17)	0.0309 (19)	−0.0021 (13)	0.0044 (15)	−0.0037 (14)
C11	0.0299 (19)	0.0279 (18)	0.044 (2)	0.0012 (15)	0.0082 (17)	−0.0040 (17)
C12	0.034 (2)	0.0268 (18)	0.040 (2)	0.0015 (15)	0.0088 (17)	−0.0029 (16)
C13	0.040 (3)	0.073 (4)	0.057 (3)	−0.001 (2)	0.016 (2)	−0.027 (3)
C14	0.037 (2)	0.046 (3)	0.062 (3)	−0.0148 (19)	0.017 (2)	−0.017 (2)
N1	0.0272 (15)	0.0255 (15)	0.0365 (18)	−0.0016 (12)	0.0054 (14)	−0.0046 (13)
N2	0.0315 (16)	0.0253 (15)	0.0326 (17)	−0.0011 (12)	0.0052 (14)	−0.0024 (13)
O1	0.0426 (17)	0.0464 (18)	0.057 (2)	−0.0065 (14)	0.0121 (15)	−0.0261 (16)
O2	0.0423 (18)	0.064 (2)	0.079 (3)	−0.0128 (17)	0.0269 (19)	−0.042 (2)
O3	0.0478 (19)	0.0438 (18)	0.061 (2)	−0.0139 (15)	0.0139 (16)	−0.0174 (16)
O4	0.0409 (18)	0.0461 (17)	0.0514 (19)	−0.0123 (13)	0.0162 (15)	−0.0021 (15)
O5	0.0487 (18)	0.0426 (17)	0.0334 (16)	0.0028 (14)	0.0141 (14)	−0.0007 (13)
O6	0.0505 (19)	0.0400 (17)	0.0382 (18)	0.0109 (13)	0.0152 (15)	0.0012 (13)
O7	0.105 (3)	0.0278 (17)	0.047 (2)	0.0048 (17)	0.037 (2)	0.0062 (14)

### Geometric parameters (Å, °)

**Table:**

Cu1—O7	1.968 (4)	C8—C9	1.415 (5)
Cu1—O3	1.994 (3)	C8—H8A	0.9300
Cu1—O5	2.020 (3)	C9—H9A	0.9300
Cu1—O6^i^	2.030 (3)	C10—N2	1.346 (5)
C1—O6	1.281 (5)	C10—C12	1.486 (5)
C1—O5	1.278 (5)	C10—C11	1.513 (5)
C1—C2	1.515 (6)	C11—O2	1.236 (5)
C2—H2A	0.9600	C11—C14	1.496 (6)
C2—H2B	0.9600	C12—O1	1.228 (5)
C2—H2C	0.9600	C12—C13	1.522 (6)
C3—O4	1.262 (5)	C13—H13A	0.9600
C3—O3	1.265 (5)	C13—H13B	0.9600
C3—C4	1.508 (5)	C13—H13C	0.9600
C4—C9	1.386 (5)	C14—H14A	0.9600
C4—C5	1.433 (5)	C14—H14B	0.9600
C5—C6	1.400 (5)	C14—H14C	0.9600
C5—H5A	0.9300	N1—N2	1.305 (4)
C6—C7	1.379 (5)	N1—H1	0.94 (5)
C6—H6A	0.9300	O6—Cu1^ii^	2.030 (3)
C7—C8	1.410 (5)	O7—H7A	0.84 (4)
C7—N1	1.422 (5)	O7—H7B	0.84 (5)
			
O7—Cu1—O3	100.84 (16)	C4—C9—H9A	120.1
O7—Cu1—O5	113.86 (16)	C8—C9—H9A	120.1
O3—Cu1—O5	124.96 (15)	N2—C10—C12	112.1 (3)
O7—Cu1—O6^i^	94.12 (13)	N2—C10—C11	124.4 (3)
O3—Cu1—O6^i^	116.10 (15)	C12—C10—C11	123.5 (3)
O5—Cu1—O6^i^	102.99 (13)	O2—C11—C14	118.2 (4)
O6—C1—O5	119.2 (4)	O2—C11—C10	119.1 (4)
O6—C1—C2	121.0 (4)	C14—C11—C10	122.7 (4)
O5—C1—C2	119.8 (4)	O1—C12—C10	120.7 (4)
C1—C2—H2A	109.5	O1—C12—C13	120.6 (4)
C1—C2—H2B	109.5	C10—C12—C13	118.7 (4)
H2A—C2—H2B	109.5	C12—C13—H13A	109.4
C1—C2—H2C	109.5	C12—C13—H13B	109.5
H2A—C2—H2C	109.5	H13A—C13—H13B	109.5
H2B—C2—H2C	109.5	C12—C13—H13C	109.5
O4—C3—O3	121.6 (4)	H13A—C13—H13C	109.5
O4—C3—C4	121.2 (4)	H13B—C13—H13C	109.5
O3—C3—C4	117.2 (4)	C11—C14—H14A	109.4
C9—C4—C5	118.7 (3)	C11—C14—H14B	109.5
C9—C4—C3	117.3 (3)	H14A—C14—H14B	109.5
C5—C4—C3	123.9 (3)	C11—C14—H14C	109.5
C6—C5—C4	121.5 (3)	H14A—C14—H14C	109.5
C6—C5—H5A	119.2	H14B—C14—H14C	109.5
C4—C5—H5A	119.2	N2—N1—C7	118.9 (3)
C7—C6—C5	118.7 (3)	N2—N1—H1	120 (3)
C7—C6—H6A	120.7	C7—N1—H1	121 (3)
C5—C6—H6A	120.6	N1—N2—C10	120.3 (3)
C6—C7—C8	121.1 (4)	C3—O3—Cu1	103.6 (3)
C6—C7—N1	117.2 (3)	C1—O5—Cu1	108.4 (3)
C8—C7—N1	121.6 (3)	C1—O6—Cu1^ii^	134.8 (3)
C7—C8—C9	120.0 (4)	Cu1—O7—H7A	121 (3)
C7—C8—H8A	120.0	Cu1—O7—H7B	127 (3)
C9—C8—H8A	120.0	H7A—O7—H7B	110.9 (18)
C4—C9—C8	119.9 (3)		
			
O4—C3—C4—C9	3.1 (6)	N2—C10—C12—C13	−11.4 (6)
O3—C3—C4—C9	−177.6 (4)	C11—C10—C12—C13	168.9 (4)
O4—C3—C4—C5	−177.5 (4)	C6—C7—N1—N2	−172.2 (4)
O3—C3—C4—C5	1.8 (6)	C8—C7—N1—N2	8.7 (5)
C9—C4—C5—C6	−1.1 (6)	C7—N1—N2—C10	176.2 (3)
C3—C4—C5—C6	179.6 (4)	C12—C10—N2—N1	−178.9 (3)
C4—C5—C6—C7	0.8 (6)	C11—C10—N2—N1	0.8 (6)
C5—C6—C7—C8	0.3 (6)	O4—C3—O3—Cu1	−4.6 (5)
C5—C6—C7—N1	−178.8 (4)	C4—C3—O3—Cu1	176.2 (3)
C6—C7—C8—C9	−1.2 (6)	O7—Cu1—O3—C3	−154.1 (3)
N1—C7—C8—C9	177.9 (4)	O5—Cu1—O3—C3	76.3 (3)
C5—C4—C9—C8	0.2 (6)	O6^i^—Cu1—O3—C3	−54.0 (3)
C3—C4—C9—C8	179.6 (4)	O6—C1—O5—Cu1	−2.5 (4)
C7—C8—C9—C4	0.9 (6)	C2—C1—O5—Cu1	178.9 (3)
N2—C10—C11—O2	2.7 (6)	O7—Cu1—O5—C1	−53.0 (3)
C12—C10—C11—O2	−177.6 (4)	O3—Cu1—O5—C1	71.1 (3)
N2—C10—C11—C14	−176.0 (4)	O6^i^—Cu1—O5—C1	−153.5 (3)
C12—C10—C11—C14	3.7 (6)	O5—C1—O6—Cu1^ii^	175.8 (3)
N2—C10—C12—O1	166.9 (4)	C2—C1—O6—Cu1^ii^	−5.7 (6)
C11—C10—C12—O1	−12.8 (6)		

Symmetry codes: (i) *x*, −*y*+1/2, *z*+1/2; (ii) *x*, −*y*+1/2, *z*−1/2.

### Hydrogen-bond geometry (Å, °)

**Table:**

*D*—H···*A*	*D*—H	H···*A*	*D*···*A*	*D*—H···*A*
N1—H1···O2	0.94 (5)	1.90 (5)	2.617 (5)	132 (4)
O7—H7A···O1^iii^	0.84 (4)	1.91 (4)	2.739 (4)	168 (5)
O7—H7B···O4^ii^	0.84 (5)	2.00 (3)	2.774 (5)	153 (6)

Symmetry codes: (iii) −*x*+1, *y*−1/2, −*z*+3/2; (ii) *x*, −*y*+1/2, *z*−1/2.
